# Empowering Students in Online Learning Environments Through a Self-Regulated Learning–Enhanced Learning Management System

**DOI:** 10.3390/bs15081041

**Published:** 2025-07-31

**Authors:** Jiahui Du, Lejia Liu, Shikui Zhao

**Affiliations:** 1 School of Humanities and Social Sciences, Beihang University, Beijing 100191, China; skzhao@buaa.edu.cn; 2 Faculty of Education, The University of Hong Kong, Pok Fu Lam, Hong Kong SAR, China; liulejia@connect.hku.hk

**Keywords:** self-regulated learning, online learning, blended learning, learning management system

## Abstract

Self-regulated learning (SRL) has been widely recognized as a critical skill for academic success in online and blended learning contexts. However, many students experience difficulty in effectively applying SRL strategies in the absence of structured instructional guidance. To address this challenge, this study developed and implemented a learning management system integrated with SRL support (SRL-LMS), specifically designed for the online component of a blended learning course. The SRL-LMS consisted of two sections: a conventional course content section and a SRL training section designed to support students in applying SRL strategies. A quasi-experimental design was adopted with 69 college students assigned to either an experimental group, with access to both course and SRL sections, or a control group, which accessed only the course section. Results indicated that students in the experimental group reported higher levels of self-regulation and showed more frequent and diverse application of SRL strategies compared to the control group. In terms of academic performance, the experimental group performed significantly better than the control group on the first exam, though no significant difference was observed on the second exam. These results highlight the effectiveness of structured SRL interventions within digital learning platforms for improving students’ self-regulatory behaviors. Future implementations should address cognitive load and incorporate strategic approaches to sustain student motivation. This study advances current SRL intervention designs and offers valuable insights for educators and instructional designers aiming to integrate targeted SRL supports in online and blended learning environments.

## 1. Introduction

As global demands for lifelong learning continue to rise, learners must develop the ability to manage and direct their own learning to thrive (Ateş, 2024). Self-regulated learning (SRL) is defined as the capacity to monitor and control one’s learning processes to achieve learning goals (Zimmerman, 2000). SRL has long been recognized as a key factor in promoting effective learning and academic achievement (Afzaal et al., 2023). In recent years, interest in SRL has intensified, particularly in response to the growth of online and blended learning environments (Jin et al., 2023; Khalil et al., 2022). Unlike traditional classroom settings where teachers structure learning paths and guide learning processes, online learning environments require students to take greater responsibility for regulating their learning. They need to sustain motivation, manage time effectively, and apply appropriate learning strategies (Perez-Sanagustin et al., 2020). This learning mode places higher demands on students’ SRL abilities (Kizilcec et al., 2017; Wong et al., 2019). However, research shows that many students lack proficiency in SRL, particularly in online contexts (Cristea et al., 2023; Jansen et al., 2020; Viberg et al., 2020). Rasheed et al. (2020) conducted a systematic review of challenges in blended learning, and they identified difficulties with SRL and the use of learning technologies as key barriers to student success in online settings.

With growing recognition of SRL as a key skill in technology-mediated learning environments, an increasing number of studies have explored ways to support students’ SRL in online and blended settings. However, existing efforts reveal limitations in both design and implementation. Specifically, current SRL interventions often lack strong theoretical foundations, fail to address all phases of the SRL cycle, and make insufficient use of students’ online behavioral data (see Section 2.2 for more details). To address these gaps, we developed a learning management system integrated with SRL support (SRL-LMS), which aimed at supporting students’ SRL in the online component of a blended learning course. The system was designed based on Zimmerman’s (2002) cyclical model and Pintrich’s (1999) model of SRL, with learning activities explicitly structured to follow the theoretical framework. In addition to covering all three phases of the SRL cycle, this study also placed emphasis on helping students learn and apply a variety of learning strategies regarding Pintrich’s (1999) model. The effective use of SRL strategies is central to fostering SRL skills and improving academic performance (Boulahmel et al., 2023; Jin et al., 2023).

To examine the effects of the proposed SRL intervention, this study addresses the following research questions:

**Research question 1:** How does the SRL-LMS affect students’ self-reported level of SRL compared to a conventional approach?

**Research question 2:** How does the SRL-LMS affect students’ self-reported use of SRL strategies compared to a conventional approach?

**Research question 3:** How does the SRL-LMS affect students’ learning performance compared to a conventional approach?

## 2. Literature Review

### 2.1. Self-Regulated Learning (SRL)

Zimmerman (2000) defined SRL as the ability to adopt appropriate learning strategies in pursuit of personal goals. In this process, students act as active participants who initiate and monitor their own learning, even in the absence of teacher guidance. Because of the central role students play in managing their own learning, SRL has long been regarded as a critical factor in academic success (Kizilcec et al., 2017). SRL is critical not only for achieving academic success but also for supporting lifelong learning, as individuals must continually adapt to new knowledge and evolving learning environments (Shih et al., 2010). The recognized importance of SRL has prompted extensive research efforts to propose and refine theoretical frameworks describing its processes and key components.

Panadero (2017) reviewed six major models of SRL and identified those developed by Zimmerman and Pintrich as the most influential. Their models offer a comprehensive framework for understanding SRL by covering cognitive, metacognitive, and resource management strategies throughout the three learning phases: forethought, performance, and reflection. Zimmerman (2002) conceptualized SRL as a cyclical process consisting of three phases that occur before, during, and after learning. This model guides educators to design instructional approaches that integrate appropriate SRL strategies for different learning stages. In the forethought phase, learners prepare for upcoming tasks by setting goals, planning, activating prior knowledge, and strengthening motivational beliefs. During the performance phase, learners monitor their learning progress and maintain engagement through strategies such as time management and self-observation. After completing learning tasks, the reflection phase prompts learners to evaluate their performance and use the insights gained to inform future learning efforts. These three phases illustrate the cyclical and dynamic nature of SRL. Pintrich (1999) categorized SRL strategies into three key dimensions. Cognitive strategies involve techniques for processing information, such as rehearsal for memorization or elaboration and organization for deeper comprehension. Metacognitive strategies refer to the regulation of cognitive processes, including planning, monitoring, and adjusting learning behaviors. Resource management strategies focus on controlling external aspects of the learning process, such as managing time and effort, creating supportive learning environments, and seeking help when necessary.

### 2.2. Tools for Supporting Self-Regulated Learning

Self-regulated learning (SRL) is widely recognized as a critical skill for academic success, as it enables students to take control of their learning processes. Numerous studies have shown that training students in SRL strategies leads to improved learning behaviors and academic outcomes (Çebi & Güyer, 2020; Pedrotti & Nistor, 2019; van Alten et al., 2020). In recent years, increasing attention has been given to the role of technology in supporting SRL across various learning contexts.

Recent studies have explored the potential of dashboards to support key SRL processes. Afzaal et al. (2023) developed an AI-based dashboard that recommended learning tasks such as assignments and lecture activities, to guide students in selecting appropriate learning actions. Yoon et al. (2021) designed a progress-tracking dashboard that allowed students to visualize their performance over time and reflect on their learning. Similarly, Cobos (2023) introduced a learning analytics system that provided feedback through dashboards, which helped students monitor their progress and make timely adjustments. Another group of studies has explored how conversational agents and AI applications can assist students in managing their learning more effectively. For example, Maldonado-Mahauad et al. (2022) created a chatbot that offered tailored suggestions related to resource use and time management. Jin et al. (2023) developed a set of AI application storyboards that explained the phases of SRL, helping students understand how to apply specific strategies. In addition to tool development, some studies have examined how the learning environment itself influences SRL. Radović et al. (2024) compared environments with different levels of SRL support and found that students receiving more structured guidance participated more actively in learning tasks. Teich et al. (2024) focused on supporting learners’ resource management skills in online courses and found that adaptive interventions significantly improved students’ ability to structure their learning environments. Dever et al. (2024) integrated prompts and feedback into an intelligent tutoring system and found that students used SRL strategies more frequently when the support aligned with their learning goals.

Prior reviews have systematically examined a wide range of SRL interventions and identified several key gaps in their design and implementation. First, the theoretical underpinnings used to guide the design of SRL interventions were often insufficiently articulated. Álvarez et al. (2022) reviewed 42 studies, including 25 SRL support tools, and found that many did not specify which SRL model informed the design of the tool. This lack of theoretical clarity limits both the coherence of the interventions and the ability to evaluate their effectiveness. Similarly, Prasse et al. (2024) analyzed 31 studies on the design of computer-assisted SRL supports and emphasized the need for stronger theoretical alignment. They argued that future research should more clearly articulate the SRL frameworks that guide intervention design and analysis. Second, many interventions fail to address the entire SRL cycle, which restricts the development of comprehensive SRL skills. While SRL involves a dynamic process that includes forethought, performance, and reflection, existing studies tend to focus disproportionately on one particular phase. Heikkinen et al. (2023) reviewed 56 studies on learning analytics (LA) interventions and found that few supported all phases of SRL. Similarly, Edisherashvili et al. (2022) examined 38 SRL interventions and reported that most concentrated on the performance phase, with limited attention given to planning or reflection phase. Third, real-time behavioral data are underexposed in both measuring SRL and delivering fundamental support. Araka et al. (2020) reviewed SRL tools within e-learning platforms and noted the absence of a conceptual framework for integrating educational data mining (EDM) into LMS-based interventions. Viberg et al. (2020) analyzed 54 studies using LA to support SRL in online learning environments and concluded that many interventions focus solely on tracking student behavior without offering guidance on how learners should respond. They argued that generating recommendations based on behavioral data is critical, as analytics alone do not automatically lead to improved learning outcomes.

To address the gaps identified in previous SRL intervention designs, this study developed a learning management system (SRL-LMS) grounded in Zimmerman’s (2002) and Pintrich’s (1999) model of SRL. The system was structured to support all three phases of the cyclical process of SRL: forethought, performance, and self-reflection. For each phase, specific SRL strategies were defined and integrated into the design of corresponding learning activities to ensure conceptual alignment and instructional coherence. To support students in monitoring and regulating their learning more effectively, the system integrated behavioral data and delivered continuous real-time feedback through a visualized spider chart (see Section 3.2 for details). These designs address the key limitations identified in prior research by offering a theoretically grounded intervention that supports all phases of SRL and incorporates real-time behavioral data to both measure students’ use of SRL strategies and provide targeted support.

## 3. Methods

### 3.1. Contexts and Participants

This study was conducted within a college-level mathematics course at a large public university in China. Participants were undergraduate students from the Faculty of Management enrolled in a compulsory semester-long course. Students were divided into two classes that used the same course materials and were taught by the same instructor, who was not informed of the group assignment. A quasi-experimental design was employed to examine how the intervention affected students’ self-reported SRL level, strategy use and academic performance. To do so, the two classes were randomly assigned to either the control or experimental group. The control group had access only to the system’s course section, which provided regular course materials. The experimental group had access to both course section and the SRL section designed to support SRL related activities. In total, the study included 69 participants, with 35 in the control group and 34 in the experimental group. All students enrolled in the course were invited to participate in the study by voluntarily signing informed consent forms, which were approved by the university’s Institutional Review Board. The consent forms clearly outlined participants’ rights, including the ability to withdraw from the study at any time and the right to review and request the deletion of collected data. The researchers collected all signed consent forms from the students prior to the start of the study.

### 3.2. Research Design

**Figure 1:**
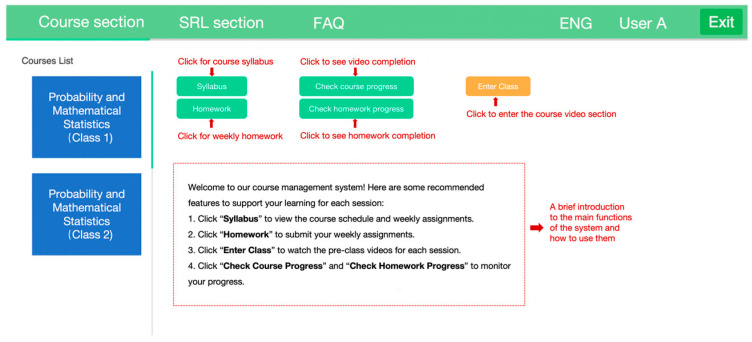
Interface of the course section of the SRL-LMS.

A learning management system integrated with SRL support (SRL-LMS) was developed to facilitate instruction and implement the interventions for this study. Students in both the control and experimental groups were required to register individual login accounts. To ensure data privacy, students registered in the LMS using their student IDs, which allowed access only to their assigned course section. Identifiable information was accessible exclusively to the researchers in this study and used only for research purposes. The system included two primary sections: a course section and an SRL section (Figure 1). All students had access to the course section, which contained instructional materials such as a course syllabus, weekly pre-class video lectures, post-class exercises, course learning materials (e.g., handouts, presentation slides), and assignment submission portals, all of which are standard features in most LMS. The major distinction between the two groups was that only students in the experimental group had access to the SRL section.

**Figure 2:**
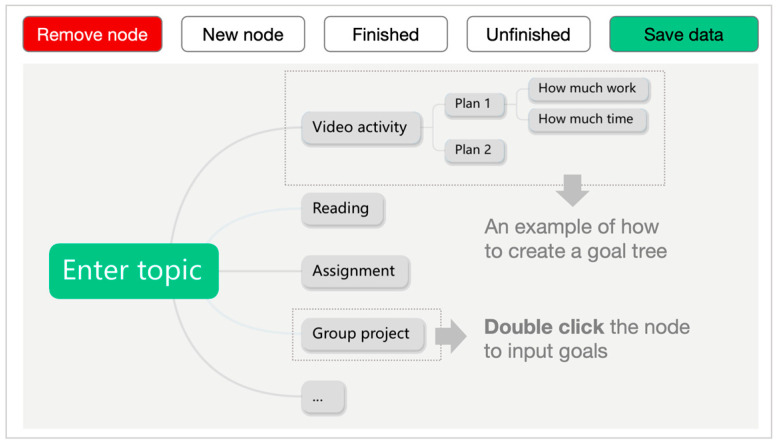
Illustration of the goal map tree in the SRL section.

The SRL section was presented as a separate component and was not integrated with the course’s learning materials. The design of the SRL section was informed by Zimmerman’s (2002) model of SRL, which outlines three cyclical phases: forethought, performance, and reflection. To support the forethought and reflection phases, a goal map tree was implemented to guide students in setting learning goals and reflecting on their progress. Each week, students were required to create learning goals prior to the start of the session. A pre-structured goal map tree was embedded in the SRL section to facilitate this process (Figure 2). The goal map tree was developed in accordance with the SMART framework (i.e., Specific, Measurable, Attainable, Realistic, and Time-bound) to guide students in formulating effective goals (Doran, 1981). For instance, rather than simply stating “watch pre-class videos” as a goal, students were encouraged to specify the time and effort they intended to allocate for the task. At the end of each week, students were asked to review their goal map tree to evaluate their progress and report the completion rate of their goals. This reflective activity reinforced the cyclical nature of the SRL process by linking goal setting (forethought) with evaluation (reflection).

**Figure 3:**
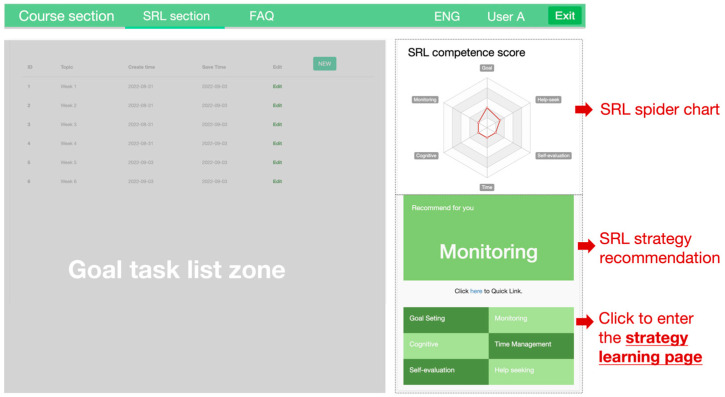
Interface of the spider chart in the SRL section.

The performance phase aimed at engaging students in actively applying various SRL strategies throughout the learning process. A real-time spider chart was implemented to provide continuous feedback on students’ use of SRL strategies. The spider chart highlighted areas where students under-performed in their use of SRL strategies and offered targeted recommendations for further practice. Specifically, the spider chart featured six SRL strategies, each represented as a dimension. Students’ engagement with these strategies was quantified by strategy scores, which were calculated and updated in real time based on their performance in SRL-related activities within the system (Figure 3). These scores were automatically calculated and updated in real time based on students’ performance in SRL-related activities (Figure 3) and were used to generate personalized recommendations. The scores can be obtained in two ways. First, students earned points by completing designated SRL strategy practices. Under the spider chart, each strategy is designed as a clickable link, which could direct students to a corresponding strategy practice page. The SRL strategy practice page consisted of two sections: the left panel provided a description of the strategy, while the right panel contained a quiz designed to assess students’ understanding of the theoretical concepts. The quiz consisted of 30 multiple-choice questions, developed by the researchers based on established SRL theories and frameworks (Pintrich, 1999; Zimmerman, 2002). Students were allowed unlimited attempts, with the system recording their highest score. This score was then reflected in real time on the spider chart and contributed to the overall SRL strategy score for the corresponding dimension.

Another way to earn SRL strategy scores was by performing specific SRL behaviors tracked by the system. These behaviors were identified by the researchers based on established SRL frameworks and prior studies (e.g., Du et al., 2023; Fan et al., 2021; Jansen et al., 2020; Matcha et al., 2020; Siadaty et al., 2016). For example, accessing the course syllabus or adding new goals in the goal map tree were considered indicators of goal setting and planning, as these actions demonstrate students’ engagement in organizing and preparing their learning activities. Table 1 displayed the behavioral indicators associated with each SRL strategy. To summarize, students could earn points by either performing these identified behaviors or completing the corresponding SRL strategy practices, with a maximum of 10 points available for each strategy. Strategy scores were updated in real time, and the system generated tailored recommendations based on students’ performance across SRL dimensions.

### 3.3. Research Procedure

The intervention period spanned 12 weeks and comprised 10 weekly learning sessions. Each session included pre- and post-class online sections and a two-hour face-to-face in-class section. The first two weeks constituted the university’s official add and drop period, during which students could freely enroll in or withdraw from the course. These initial weeks were therefore used to familiarize students with the course structure and the learning management system, including how to access materials and complete assigned tasks. The formal intervention began in week 3. During this week, students in both the control and experimental groups completed the pre-SRL test and the pre-knowledge test. They were also informed of the weekly requirement to participate in both pre- and post-class online activities. The first exam took place in week 9. Later in week 14, students from both groups completed the second exam, post-SRL questionnaire, and an open-ended survey.

### 3.4. Data Collection and Analysis

To comprehensively explore the impact of SRL-LMS on students’ self-reported SRL level and learning performance, both quantitative and qualitative data were collected. Specifically, we employed three primary measures: the SRL tests, which assessed students’ self-reported SRL level before and after the intervention; an open-ended survey, which allowed students to articulate their use of SRL strategies and reflect on their learning experiences; and two formal course exams, which measured students’ academic performance in relation to the course content.

To address the first research question, we employed the Motivated Strategies for Learning Questionnaire (MSLQ; Pintrich et al., 1991), a widely recognized instrument in self-regulated learning (SRL) research with well-established reliability and validity. The MSLQ consists of 81 items across two sections: motivation and learning strategies. For the purpose of this study, we focused exclusively on the learning strategies section, which includes 50 items. These items are divided into three subscales: cognitive strategies (19 items), metacognitive strategies (12 items), and resource management strategies (19 items). McDonald’s omega (ω) was calculated to assess the internal consistency of each MSLQ subscale. The reliability coefficients were 0.88 for cognitive strategies, 0.81 for metacognitive strategies, and 0.82 for resource management strategies. These values indicate good to excellent internal consistency and provide evidence that the instrument was reliable for the current study. Students in both groups were asked to report their SRL performance on a seven-point Likert scale ranging from “not at all true of me” to “very true of me.” The quantitative data obtained from the questionnaire were analyzed using IBM SPSS Statistics software 28.0. Specifically, we first conducted a one-way analysis of covariance (ANCOVA) to assess the overall self-reported SRL level of students in each group after the intervention, using their pre-test SRL scores as a covariate. Then, we conducted Mann–Whitney U tests to further examine the differences in each SRL subscale between the control and experimental groups.

For the second research question, we conducted an open-ended survey to explore students’ use of various SRL strategies throughout the course. The aim was to provide students with an opportunity to explicitly demonstrate their SRL practices, and their responses served as a supplement to the SRL scale data. The sample questions in the survey are “Please describe how you typically study for this course” and “What learning strategies did you use during the learning in the course and how did you use them?”. Students’ responses were collected and manually coded by the researchers. The coding scheme was determined by the researchers according to the definitions of each SRL strategy by Pintrich (1999). Based on the theoretical definitions, researchers identify key words and phrases in students’ descriptions of their learning experience and process. Two researchers independently coded all responses, and the first researcher randomly selected 20% of the responses from each group for reliability checking. The inter-coder agreement rate was 93%, and the Cohen’s Kappa value was 0.82, which indicated substantial agreement between coders. Any disagreement was resolved by discussions between the two researchers.

To assess student learning performance in the third research question, we conducted a pre-knowledge test and two formal exams. All tests and exams were designed and graded by the course instructor, who has over 20 years of teaching experience in the field. To ensure that students in the control and experimental groups had similar levels of mathematical knowledge, we conducted a pre-knowledge test. The test consisted of ten questions, including six multiple-choice and four true/false questions. Each item was worth 10 points, and the total score was 100 points. Students also completed two formal exams during the semester. The first exam was held at midterm, and the second took place in the final week of the course. Each exam included five open-ended problem-solving questions on topics in probability and statistics. The maximum score for each exam was 100 points. Scores were given based on the accuracy and completeness of students’ responses. Exam 1 covered content from Chapters 1 and 2. Exam 2 focused on material from Chapters 3 to 5. Data from these assessments were analyzed using SPSS Statistics software. A series of Mann–Whitney tests were performed to examine score differences between the two groups on the pre-knowledge test and two exams.

## 4. Results

### 4.1. Students’ Self-Reported SRL Level

We first examined students’ self-reported SRL level based on their scores from the Motivated Strategies for Learning Questionnaire (MSLQ). A one-way ANCOVA was conducted to compare post-SRL test scores with pre-SRL test scores entered as a covariate to control for initial group differences. All preliminary assumptions were tested and met before proceeding with the ANCOVA. The Shapiro–Wilk test confirmed that post-SRL test scores were normally distributed in both groups, W (35) = 0.986, *p* = 0.920 for the control group, W (34) = 0.951, *p* = 0.133 for the experiment group. The assumption of homogeneity of regression was satisfied, F (1, 65) = 1.509, *p* = 0.224. Levene’s test confirmed that the assumption of homogeneity of variance was met, *p* = 0.986. The assumption checks confirmed that ANCOVA was appropriate for analyzing differences in students’ post-SRL test scores. After adjusting for pre-SRL test scores, a statistically significant difference was found in the post-SRL test scores between the control group (Madjusted = 4.80, SE = 0.08) and the experimental group (Madjusted = 5.04, SE = 0.08), F(1, 66) = 4.65, *p* = 0.035, η2 = 0.066. To further examine group differences in specific dimensions of the post-SRL test, Mann–Whitney tests were conducted at the subscale level. The Mann–Whitney test was adopted because the Shapiro–Wilk test indicated that the SRL subscale scores were not normally distributed. The results showed that the experimental group scored significantly higher than the control group on each subscale. For the cognitive subscale, the median score was 4.68 in the control group and 5.34 in the experimental group, U = 377.5, *p* = 0.009. For the metacognitive subscale, the median score was 4.58 in the control group and 5.29 in the experimental group, U = 366.5, *p* = 0.006. For the resource management subscale, the median score was 4.68 in the control group and 5.05 in the experimental group, U = 389.5, *p* = 0.014 (Table 2 and Table 3).

### 4.2. Students’ Self-Reported Use of SRL Strategies

An open-ended survey was conducted to explore students’ self-reported use of SRL strategies during the course. The survey included two specific questions: “Please describe how you typically study for this course” and “What learning strategies did you use during the learning in the course and how did you use them?” Students were encouraged to respond freely and describe their learning experiences and behaviors in detail. The researchers then coded the responses to identify the SRL strategies interpreted in each response. A single response could be linked to multiple strategies if it aligned with the conceptual definitions. For each group, the number of students who reported using specific strategies was recorded, along with the proportion of the total strategies identified (Table 4).

A total of 46 students reported the cognitive strategies they used while studying for the course, including 21 students from the experimental group and 25 from the control group. Drawing on Pintrich’s (1999) framework, three key cognitive strategies were used to analyze their responses: rehearsal (e.g., repetition or recitation), elaboration (e.g., paraphrasing or summarizing content), and organization (e.g., structuring or connecting ideas). Most of the strategies reported by students involved taking notes, summarizing materials, or memorizing key concepts introduced in the course. Examples of student responses include the following:

*“In class, I take notes on key points and important details mentioned by the teacher. After class, I review and memorize the necessary content, reinforce it through consolidation, and complete related exercises.”*

*(Student No. 1, EG)*

*“For each class, I take notes, complete assignments, and memorize key formulas. Before exams, I summarize key topics and common question types.”*

*(Student No. 5, CG)*

A total of 32 students described the metacognitive strategies they employed while studying for this course, including 18 from the experimental group and 14 from the control group. Based on Pintrich’s (1999) framework, metacognitive regulation encompasses strategies such as planning, monitoring, and regulating one’s learning processes. In their responses, students referred to behaviors such as previewing content and setting study goals before beginning a session, checking their understanding through self-assessment, and regularly reviewing course material after class. The following examples illustrate these reported strategies:

*“After studying, I categorize the content into potential test points and complete exercises based on these points, typically every two chapters. Through these exercises, I identify problem-solving strategies and assess how well I understand different types of math problems.”*

*(Student No. 11, EG)*

*“Usually, about a week before a test, I review the lecture slides and complete exercises from both the slides and the textbook to check my understanding. Then, I revisit key concepts that are likely to appear on the test to reinforce my memory.”*

*(Student No. 19, CG)*

In total, 37 students reflected on how they managed their study resources throughout the course, with 22 from the experimental group and 15 from the control group. Resource management strategies typically involve time management, structuring the learning environment, and seeking help when needed (Pintrich, 1999). In their responses, students described establishing consistent study routines and designated learning spaces, as well as turning to teachers, peers, or online resources for support when facing difficulties. The following examples illustrate these reported strategies:

*“Except for following the teacher during class, I make use of online learning resources and preview the textbook the night before each session. I prefer to study in the dormitory’s quiet study room.”*

*(Student No. 3, EG)*

*“I work through the exercises at the end of the textbook and the examples provided in the lecture slides to organize the key concepts. When I encounter problems I don’t understand, I ask my classmates or roommates for help.”*

*(Student No. 14, CG)*

### 4.3. Students’ Learning Performance

We examined the learning performance of students in both groups. We first conducted a pre-knowledge test to examine whether a significant difference existed between the control group and the experimental group, with a maximum score of 100 points. The Shapiro–Wilk test indicated that the pre-knowledge test scores for both groups were non-normally distributed, thus we conducted Mann–Whitney test. The results showed that there were no initial differences on prior knowledge levels of both groups, U = 486.00, *p* = 0.177. Then, we examined students’ learning performance on the two exams. Each exam included five math questions related to the topics discussed in the course, with a maximum score of 100 points. Due to the fact that the Shapiro–Wilk test showed that the scores of two exams were non-normally distributed, we conducted Mann–Whitney tests. The results of Exam 1 showed significant results between two groups, Median = 70.00 for control group, Median = 87.50 for experiment group, U = 421.00, *p* = 0.036. The results of Exam 2 indicated no significant differences on students’ learning performance, with Median = 74.00 for control group, Median = 70.00 for experiment group, U = 559.50, *p* = 0.670 (Table 5).

## 5. Discussion and Implication

To address the first research question, we examined students’ self-reported SRL level using pre- and post-SRL test scores. Students in the experimental group reported significantly higher post-SRL test scores compared to those in the control group. A closer analysis of the subscales showed that the experimental group outperformed the control group across all cognitive, metacognitive, and resource management dimensions. These results suggest that the SRL-LMS had a significant positive effect on students’ self-reported level of SRL. As mentioned before, the key distinction of the LMS developed in this study is the inclusion of an SRL training section explicitly designed to cultivate SRL strategies. Engaging with these training activities likely contributed to the experimental group’s superior performance across all SRL dimensions. Our findings are consistent with prior studies that demonstrate targeted SRL interventions (i.e., digital scaffolds and structured supports) can effectively enhance students’ SRL abilities (e.g., Yoon et al., 2021; Shyr & Chen, 2018; Wong et al., 2019). These results highlight the importance of integrating adaptive scaffolding mechanisms into digital learning environments to foster SRL more effectively (Lim et al., 2022). Future research should further explore the design and implementation of structured SRL training modules that not only supplement academic content but also actively engage students in developing and applying SRL strategies. Such modules may be structured as a separate SRL section, as demonstrated in this study, or embedded within course content and activities. The key is to ensure that support for SRL is not undermined by an exclusive emphasis on course content. It is also important to track students’ engagement with SRL interventions and their use of specific strategies over time. This enables instructors to provide timely and targeted guidance, which is particularly important given that students may lack the expertise to monitor and adjust their use of SRL strategies on their own.

To address the second research question, we analyzed students’ responses to an open-ended survey to examine their use of SRL strategies. The results indicated that nearly half of the control group students relied primarily on a single type of strategy, most often cognitive strategies, whereas students in the experimental group demonstrated a more balanced and frequent use across all three categories. This difference may be attributed to the SRL section integrated into the learning system used by the experimental group, which introduced students to a broader set of strategies and encouraged more flexible application. The use of varied strategies may also indicate a higher level of SRL competence, as the employment of a variety of strategies tends to be related with more adaptive self-efficacy, interest, and task value (Callan et al., 2022). The ability to adapt by adjusting, replacing, or discarding ineffective approaches is a key aspect of successful SRL learners (Zimmerman, 2002). This suggests that future SRL designs should not assume that learners are already equipped with a full understanding of SRL strategies. Instead, instruction should incorporate the explicit introduction of diverse strategies, along with structured opportunities for students to apply them in practice. By doing so, students are encouraged to move beyond reliance on familiar or routine strategies and develop more adaptive approaches of regulating their learning.

Results from the second research question indicated that students in the experimental group reported slightly higher frequencies of using metacognitive strategies compared to the control group. This is consistent with previous findings that learners who receive personalized scaffolding are more likely to engage in metacognitive activities than those who do not (Lim et al., 2022). Compared to cognitive strategies, metacognitive skills are more complex and typically require deliberate instructional support for effective development. Previous research suggests that students benefit from tailored assistance, as these activities do not typically emerge without guidance (Nussbaumer et al., 2011). Prior evidence also indicates that students who received adaptive metacognitive prompts along with feedback reported using SRL strategies more frequently than those who did not receive such support (Lee et al., 2010). This suggests that future research should build on the concept of structured SRL training by placing greater emphasis on metacognitive skill development, particularly through the design of adaptive metacognitive activities and the provision of timely feedback, in order to enhance students’ motivation and engagement in SRL.

The third research question examined differences in learning performance between the two groups. The results showed that the experimental group scored significantly higher than the control group in Exam 1, while no significant difference was found in Exam 2. This mixed outcome aligns with prior research, which has also reported inconsistent effects of SRL interventions on learning outcomes. Viberg et al. (2020) reviewed 54 studies on the use of learning analytics (LA) to support SRL in online learning environments and found that only 20% reported improvements in learning outcomes. One possible explanation for these varied findings lies in the diversity of performance measures used across studies. In this study, exam scores were used as the measure of learning performance. Similarly, van Alten et al. (2020) employed course-based learning outcome test and found no significant differences between students who received SRL prompts and those who did not. Studies using alternative performance metrics have also yielded mixed results. For example, Yoon et al. (2021) measured student performance through weekly quiz scores and video completion rates and found that students who used an SRL-support dashboard outperformed those who did not. Another study by Perez-Sanagustin et al. (2020) compared final course grades, which included various graded components such as exams and participation, and they found no significant differences between the groups. Some studies have reported that SRL interventions could improve performance in certain tasks but not in others. Teich et al. (2024), for example, provided adaptive SRL interventions and reported that the experimental group outperformed the control group on only two of the six quizzes.

Another notable result from the third research question is the reversal in performance between groups across the two exams. While the experimental group scored significantly higher in Exam 1, the control group outperformed them in Exam 2. One possible explanation is that the combined effect of the cognitive demands from the SRL interventions and the increased workload in the final weeks of the semester placed additional strain on students in the experimental group. Although both groups experienced increased academic pressure during the final weeks, students in the experimental group had the extra cognitive demands from using the SRL interventions. In contrast, students in the control group used conventional study methods that did not require extra cognitive effort. This combination of factors could explain why the experimental group’s performance declined on Exam 2 compared to Exam 1, while the control group maintained more stable performance. This interpretation could be supported by Lim et al. (2022), who reported no significant differences in learning outcomes between intervention and non-intervention groups and attributed the result to increased cognitive load among students receiving scaffolds. Bellhäuser et al. (2016) also observed that although all groups improved in mathematics knowledge, the control group demonstrated a larger increase than the experimental groups. The authors explained this outcome as resulting from the time demands of the web-based SRL training, which may have reduced the time available for practicing mathematical problems. These findings suggest that future research should consider adding follow-up tutorials or training sessions to reduce cognitive overload and guide students to use SRL tools in a more time-efficient and manageable way. Another plausible explanation for the decline in performance is that students may initially engage with new tools but gradually lose motivation to continue conducting SRL. This concern has also been raised in earlier studies. For example, Kizilcec et al. (2020) found that the effect of plan-making prompts was limited to the initial weeks of the intervention. These results point to the need for future research not only to design effective SRL scaffolds but also to explore strategic approaches for sustaining student motivation over time. As Nussbaumer et al. (2014) noted, the development of SRL is a long-term process. The full benefits of SRL scaffolds are unlikely to be evident within short-term interventions. Therefore, future work should focus on extended training programs that provide continuous support for both SRL strategy use and motivational engagement. For example, instead of keeping the intervention unchanged throughout the learning process, future research could examine how to adjust the level of support in response to students’ SRL performance and engagement. When students perceive that support is responsive to their needs, they may feel better supported and become more motivated to stay engaged throughout the learning experience.

Despite these contributions, several limitations should be acknowledged. One limitation of this study is the limited range of SRL trace data captured by the online system, which was restricted by the design of the course’s instructional activities. As a result, the collected data was not included in the analysis. Future research should consider more comprehensive methods for collecting and analyzing online behavioral data. Such data can complement self-report measures and offer deeper insights into students’ SRL processes. Implementing similar designs in fully online courses may also allow for richer data collection and more detailed examination of students’ SRL behaviors. Additionally, since this study relied on self-reported data, it remains unclear to what extent students actually engaged in the activities. This limitation reduces our ability to assess how variations in engagement may have affected learning outcomes. Future research should consistently track student engagement throughout the course using behavioral data to allow for a more detailed analysis of the relationship between engagement patterns and academic performance. Moreover, the small sample size in this study reduces the ability to generalize the findings to broader student populations. Future research should involve larger, more diverse samples to enhance the validity of the findings across varied educational settings. Finally, the intervention period was limited to a ten-session course, which may have constrained the long-term development of SRL skills. We recommend extending the duration of future research to examine sustained engagement and outcomes over time.

## 6. Conclusions

This study examined a new approach to facilitate students’ self-reported SRL strategy use within the online component of a blended learning context. To this end, the study developed a learning management system integrated with SRL support (SRL-LMS) to guide students in applying a range of SRL strategies. The results demonstrated the potential of structured SRL training to enhance both students’ self-reported SRL level and use of SRL strategies. This study contributes to the literature in the following ways. First, this study addresses the gaps identified in previous research by adopting an integrated solution of supporting SRL rather than tackling isolated issues. Informed by theoretical, structural, and data-driven considerations, the system developed in this study offers a comprehensive response to the limitations of existing SRL interventions. Second, this study introduces an innovative system design that extends beyond the conventional functions of typical LMS-based interventions. While most existing tools focus on recommending course-related tasks and promoting task completion, our system is explicitly designed with the development of self-regulation as its core objective. By doing so, this study advances current SRL scaffold designs and introduces a novel system that offers practical insights for improving learning support in online and blended environments.

## Figures and Tables

**Table 1:** Indicators of SRL strategies identified in the system.

SRL Strategy	Description in SRL Frameworks	Behavioral Indicators
Goal setting and planning	Setting learning goals and planning for future learning	Access course syllabus; create a new goal in the map tree; edit goals in the map tree; complete goal setting and planning strategy quiz
Monitoring	Monitoring comprehension and learning progress	Click on the progress bar to check learning progress; edit goals in the map tree; answer the monitoring prompt question; complete monitoring strategy quiz
Cognitive strategy	Reciting and elaborating the learning content	Watch one video twice or more; answer the cognitive prompt question; submit reflection paper; complete cognitive strategy quiz
Time management	Managing and controlling the learning time	Answer time management prompt question; create a new goal for the upcoming week; report completion rate of the goals in the map on time; complete time management strategy quiz
Self-evaluation	Checking learning progress and performance	Click the progress bar to check learning progress; report completion rate of the goals of the map tree; watch one video twice or more; complete self-evaluation strategy quiz
Help-seeking	Seeking help from others or finding answers via external resources	Access FAQ page; access the course notification page; complete help-seeking strategy quiz

**Table 2:** SRL post-test scores of two groups.

Group	N	Mean	Standard Deviation	Adjusted Mean	Standard Error	F	*p*
EG	34	5.16	0.64	5.04	0.077	4.651	0.035
CG	35	4.69	0.73	4.80	0.076		

**Table 3:** Mann–Whitney test analysis of the SRL subscales.

Subscale	Group	N	Median	Mann–Whitney U	*p*-Value
**Cognitive** **(19 items)**	EG	34	5.34	377.5	0.009
	CG	35	4.68		
**Metacognitive (12 items)**	EG	34	5.29	366.5	0.006
	CG	35	4.58		
**Resources management** **(19 items)**	EG	34	5.05	389.5	0.014
	CG	35	4.68		

**Table 4:** SRL strategy coding scheme and summary of results.

SRL Strategy Category	Example Keywords or Phrases	EG Students (N, %)	CG Students (N, %)
Cognitive strategies	recitation, highlight, underline, paraphrase, summarize, note-taking	21 (34%)	25 (46%)
Metacognitive strategies	set goals, task analysis, track of attention, self-test, review, comprehend	18 (30%)	14 (26%)
Resource management strategies	time management, effort regulation, environment structuring, help-seeking	22 (36%)	15 (28%)

**Table 5:** Mann–Whitney U test results for the two exams.

	Group	N	Median	Mann–Whitney U	*p*-Value
Exam 1	EG	34	87.5	421.00	0.036
	CG	35	70.0		
Exam 2	EG	34	70.0	559.50	0.670
	CG	35	74.0		

## Data Availability

Data cannot be shared openly but are available on request from authors.
